# Elements of Practical Chemistry

**Published:** 1832-04-01

**Authors:** 


Elements of Practical Chemistry, comprising a Series of Experiments in every Depatment of Chemistry, with Directions for performing them, and for the Preparation and Application of the most important Tests and Reagents. By *David Boswell Reid*, Lecturer on Chemistry, &c. Octavo, pp. 511. Edinburgh, 1830. Second Ed. 1831.

Chemistry, like anatomy, ought to be studied in its practical details; it is not by reading, or by lectures alone, that the student can acquire a useful and available knowledge of either science; he must visit the laboratory and the dissecting-room, and *there* he must put his hand forth to the work; he must labour to understand all the processes of manipulation and experiment, however teasingly minute—he must acquire a neatness and facility in handling and in operating; and in all his investigations and studies, he should remember that perhaps one day, and that not far distant, he may be cited, as a responsible and professional man, to give his opinion on some important enquiry.

We have long been convinced that the mode of instruction, in almost all branches of education, and especially as relates to the young surgeon and physician, is much more of an embellishing, than of a practically useful nature; we have found it so ourselves, and doubt not that others also, when they have left the schools, and are once engaged in the vortex of the business-duties and business-cares of life, have been sorely disappointed, and deceived in some of their supposed professional acquirements. They might be able, perhaps, to discourse well and volubly on the intricacies of caloric and electricity, and be able, or, at least, imagine themselves able, to weigh the merits of the doctrines of atoms and of equivalent proportions; but, if a case of poisoning occurs in the town where they reside, or in the regiment or ship to which they are attached, then, but not generally till then, do they find that their chemical knowledge is mere parade of language, and is utterly useless in practice. We shall never forget the anxiety and fever of mind which were our lot, when, at an early period of life, in a foreign climate, we were called upon to deliver our opinion, in a court of justice, on a case which involved many appalling suspicions; and, although the testimony was declared in all respects satisfactory, well do we remember the impressive vow we made of more intimately and assiduously studying all the details of experimental investigation. We trust that we need not use many arguments to urge the attention of our younger readers, and probably also of those more advanced, to make themselves masters of every branch of science and of knowledge which has any reference to the profession, of which we are members. Did we deem it necessary to excite their appetite and zeal for honorable fame and distinction, we could tell them of more than one example of fortune and renown having been achieved by an acquaintance with experimental chemistry.

At the present moment, there is a senator, than whom none is more deservedly respected and prized by his country, whose personal history affords an instructive commentary upon our remarks. During early life, he was an assistant-surgeon in one of the regiments in India, during the administration of the Marquess of Cornwallis; the troops had been for some time engaged in bombarding a town; and it was remarked by the engineers, that the balls almost always fell short of their aim; the gunpowder was considered faulty, but the question was, how were they to ascertain this accurately? Orders were issued to all the surgeons of the regiments to analyze it, and to send a report of their trials to the Governor. Few were able to comply with the command at all; and those who made the attempt were baffled. There was, however, one youthful assistant, whose great delight at College had been the study of practical chemistry, who succeeded perfectly in the investigation, and whose well-drawn and accurate report satisfied all, that various impurities had been mixed with the saltpetre used in the manufacture of the powder. The reward due to such merit was promptly bestowed; and soon after he rose to proud and enviable eminence over his fellows.

So many have been the improvements, and so great has been the advance, of the science and art of chemistry within the last 30 years, that, from being most uncertain and conjectural, its principles and its facts, as now ascertained, bear all the stamps of demonstrative proof. Moreover, there is a beautiful yet convincing simplicity in carrying on the processes and details of analysis and combination; for the chemist no longer requires, at least for ordinary experiments, the expensive and complex apparatus of a large laboratory; a corner of his parlour will in general suffice. Provided with a blowpipe, of a size not larger than a pencil-case, and with a set of small glass tubes, which he can make for himself in less than a minute, and the requisite test-agents, which may be all contained in the compass of a writing desk, the student can pursue a course of very extended and most instructive research.

It is to the Swedish philosopher Berzelius and to our no less distinguished Wollaston that we owe so much. They have simplified and rendered easy most of the experiments of chemistry, and have brought them within the reach of every lover of science, wherever he may chance to be placed, and at whatever distance from the schools of knowledge. When, therefore, in addition to the advantages already explained, we consider that practical chemistry is not only of acknowledged importance to those who are engaged in particular professions, but is now regarded as a most valuable branch of general education, from the extent to which it can be applied in explaining the phenomena of nature, and improving the processes of art, further arguments surely are unnecessary to inculcate its high utility on the minds of our medical brethren. No members of society are expected to have a more widely diffused and accurate information on all topics than the surgeon and physician, especially when residing in a foreign climate. Wherever they may be placed, various and most important are the duties required of them; and not the least necessary is that of being efficiently acquainted with the different branches of science; for to them it is that reference is often made in an hour of difficulty and embarrassment. It is well and forcibly observed by Mr. Reid, that—

"Chemistry is now no longer confined to particular professions, arts, and manufactures, but extends its dominion over the whole economy of nature, and is seen ministering in every direction to the comforts and necessities of daily life. Wherever there are animals, vegetables, or minerals,—wherever there is earth, air, or water, its agencies are found to be in continued operation, and act an essential part in sustaining the life, order, and harmony of the whole. The power and number of its instruments, the exactnesss and precision of its analytic methods, the infinite variety of materials on which it operates, the endless combinations which it can effect, and the creative energies it exerts, have given a splendour to its progress that has arrested the attention of the world, and excited a daily increasing interest in its investigations." vi.

In our review of this work we have it not so much in design to present to our readers a detailed analysis of its contents, valuable and instructive as these certainly are, as to proclaim the great importance which we attach to that branch of education, to which it forms an excellent guide.

To those who are anxious to obtain, or to revive, the knowledge of experimental chemistry, there is no publication which we can recommend with more cordial confidence. We have very carefully examined the various processes detailed and recommended, and having either performed them ourselves or seen them performed, we can vouch for their accuracy and truth.

Mr. Reid has divided his work into two parts; the first comprising an arranged series of experiments on all the objects of chemical investigation; and the second describing and explaining the important doctrines of equivalent proportions; of electricity and galvanism; of the methods of estimating the specific gravities of bodies; also the various apparatus of furnaces, crucibles, retorts, &c.—the appropriate lutes, and cements, the different blowpipes, and the mode of using them; in short, every thing which the chemist can wish to know.

We shall select a few extracts that our readers may be enabled to judge of the style, and general character of Mr. Reid's work. The description of the effects of the nitrous oxide, or intoxicating gas is full of truth and interest.

"107. The characteristic property of this gas is the singular action which it has upon the animal economy, a high degree of excitement being produced after a few inspirations, which is accompanied by a rich and genial glow that pervades the whole frame, and the most pleasing and thrilling sensations, particularly in the chest, and a rapid succession of vivid ideas, with increased power and disposition to muscular exertion, which it is impossible for any one to restrain who has breathed freely of the gas. Out of nearly a hundred gentlemen attending my classes for practical chemistry, who have taken it within the last eighteen months, only one disliked it, complaining of a disagreeable sensation which pervaded the whole body after he had begun to inspire it, but which passed away in a minute or two, without leaving any bad effects or any disagreeable impression. In another case, the individual fainted after six or seven inspirations, but recovered with a kind of start in a few seconds, without subsequently experiencing any kind of depression. On four or five it had no apparent effect; of these, some did not take the gas properly, but one of them, a South American, took a larger dose three times in succession than all the rest, and breathed with every precaution, and after exhausting the lungs as completely as possible of atmospheric air, but still without being in the slightest degree affected by it, a circumstance that attracted our attention more particularly, as it had been lately affirmed in France that the effects which are usually ascribed to it may be traced more to the imagination than to any peculiarity in the gas itself. The rest were all affected by it in a manner which removed all doubts of the power of this singular gas, even in those who were previously impressed with the idea of the accuracy of the statements made abroad.

108. Occasionally it acted so powerfully on the system, that seven people were required to restrain a single individual while under its influence, and prevent him injuring himself or others; no person, therefore, should breathe freely of it by himself. It is curious, that the excitation which it produces passes away as speedily as it is induced, in no case that I have seen did it ever last longer than thirty-five seconds, though it often leaves a cheerfulness and gaiety of disposition for hours afterwards; nor is this state of excitation accompanied by any subsequent depression, as is the case with other stimuli, except when it has given rise to violent muscular exertion, when it is always accompanied by a corresponding degree of exhaustion.

109. Different individuals require different quantities of this gas in order to be effected by it, and by varying the quantity, the same person generally experiences all the varieties of effect which it produces, from the most gentle and agreeable stimulus, to the most furious excitement, when he will show a degree of muscular power of which it might scarcely be supposed the human frame was susceptible. In some cases, though the individual cannot restrain himself, he is perfectly conscious of his own actions and of all that is going on around him, in others nothing is recollected from the time a slight giddiness is felt till its effects pass away, when the individual often recovers with a wild start, as if previously unconscious of the place where he is, and those by whom he is surrounded. I am strongly disposed to believe from the expression of the countenance, and from individuals sometimes attempting to knock their heads on the ground, that very severe pain is sometimes felt where large doses have been taken, although, on questioning them afterwards they have invariably expressed the reverse, and affirmed that their sensations were highly pleasurable.

Those who had never previously heard of the nature of this gas, nor were aware at the time of the effect it was likely to produce, frequently stopped suddenly and exclaimed, 'that they felt as if they were lighter than the atmosphere, and were going upwards,' a sensation very generally experienced by all who breathe this gas." 45.

After having unfolded and explained the qualities, and properties of the three primary gases, oxygen, hydrogen, and nitrogen, and of the different compounds which they form with each other, the author treats of the simple non-metallic inflammables, sulphur, phosphorus and carbon ; the last is the most important, from the great number, and singular diversity of the products of its combination with other substances; as for example, the *carbonic oxide ;* the *carbonic acid,* one of nature's most important agents; the *olifiant gas,* to which the beautifully illuminating power of oil-gas is indebted; the *carburetted hydrogen* or fire-damp, so destructive in coal mines; the interesting class of medicinal substances, *alcohol and æther ;* and lastly cyanogen, or compound of nitrogen and carbon, which when united with hydrogen gas forms the hydrocyanic, or prussic acid, of all poisons the most instantaneously destroying. The physical properties of this extraordinary substance are detailed as follows.

"354. Pure and concentrated hydrocyanic acid is a limpid fluid like water, but has a strong and penetrating odour, producing severe headach, with nausea and even fainting, when the vapour which it emits is incautiously inspired. I have seen a very stout young man so much affected by smelling some diluted acid, which had been prepared three months before from the ferrocyanate of potash, by the process described, that the bottle containing it fell out of his hand, and for half an hour afterwards he was almost totally unconscious of what was going on around him. It is seldom that the diluted acid affects any one so powerfully, but this shews the great care which should be taken in making any experiments with this substance. A single drop of the strong acid, introduced into the throat of a large dog, kills it after a few hurried inspirations. Its odour is similar to that of the peach blossom, bitter almond, &c. which indeed derive their agreeable flavour from the presence of a small quanity of this acid. I have seen the diluted acid frequently used a year after it had been prepared, and its strength did not appear to be impaired, but the strong acid is sometimes decomposed in a few hours after it has been made, and can seldom be kept more than a fortnight at ordinary temperatures.

Its taste is cool at first, but it soon becomes hot and irritating. It evaporates rapidly when exposed to the air; if a drop is suspended at the extremity of a small rod, part of it is congealed by the cold produced by the evaporation of the rest." 151.

In cases of poisoning from prussic acid the medical man is frequently required to describe and explain the tests which prove its presence; and to narrate the experiments which he has performed with this view. The most delicate re-agents are the nitrate of silver, the sulphate of copper, and the pro-sulphate, or green sulphate of iron; this last, from its forming the fine pigment of Prussian blue, if caustic potash be added at the same time, is considered by far the most minutely discriminating test.

"363. Dr. Ure states, that this acid may be detected by the sulphate of iron when mixed with 10,000 parts of water, and that the sulphate of copper produces a slight milkiness in water, containing only a 20,000dth part. According to M.M. Leuret and Lassaigne, two or three days after death, it is impossible to detect this poison, as it is soon decomposed or volatilized. Where it is suspected that death has been occasioned by it, they recommend the intestines to be cut into small pieces, and put into a retort with their contents and some water, adding a small quantity of sulphuric acid and applying a gentle heat, which should not exceed 212. The volatile products are condensed in a receiver kept cold with ice, and tested in the manner we have described. The odour alone is often sufficient to indicate its presence." 154.

To counteract the effects of this most potent poison, the remedy upon which we should chiefly rely is the cold affusion.

"Dr. Herbst made a number of experiments on animals, and states that when the dose of the poison was not sufficient to prove fatal, two affusions of cold water in general removed every unpleasant symptom; when a larger dose was given, it was found necessary to repeat it more frequently, and to persevere for a considerable time. The certainty of success depends much on the early employment of the remedy. He tried liquid ammonia repeatedly, which has been much extolled as an antidote to this poison, but says that it will scarcely ever save life, where a dose sufficient to prove fatal has been given, and the symptoms have continued for some time, though he admits, that where the quantity administered is not able to destroy life, it is of great benefit in mitigating the severity of the symptoms. Another evident objection to the use of ammonia, as Dr. Herbst remarks, is, that it excoriates the parts to which it is applied, and when sufficiently diluted to be free from this inconvenience, it is of very little use. The cold water was poured freely over the head and back, and afterwards over the whole body." 153.

Another very important vegetable acid is the oxalic, which, it is well known, bears a considerable resemblance in external appearance when the crystals are small, to Epsom salts, and has frequently been sold by mistake. By the application of very simple chemical tests, these two substances may be most readily distinguished.

"Dr. Christison and Coindet, in an able memoir on poisoning by oxalic acid in the Edinburgh Medical and Surgical Journal, have shown that chalk and magnesia are certain antidotes to this poison when administered in proper time, the oxalates of lime and magnesia which are formed being quite inert." 167.

Having fully treated of the non-metallic simple bodies, the author proceeds to examine the second great class of undecomposed substances, the metals and their various compounds.

We shall allude to one only, viz. mercury and its salts; and so very comprehensive and interesting are the experiments by which this active mineral may be detected in solution, that we are induced to transfer to our pages the report of them as detailed by Mr. Reid.

"The most delicate, perhaps, is that proposed by Mr. Sylvester. A drop of the liquid suspected to contain it is to be placed on a piece of gold leaf, or any piece of solid gold, and the point of a nail or pen-knife or of any small piece of iron or zinc placed in contact with the moistened surface; if any mercury be present, the gold will immediately become white where it is touched by the other metal, uniting with the mercury and forming a solid amalgam, which retains its white colour after the fluid has been wiped off.

986. Put a piece of copper into a solution of any salt of mercury; part of the copper will be dissolved, combining with the oxygen of the oxide of mercury and the acid with which it was previously united, while an equivalent quantity of metallic mercury will be precipitated.

Add a solution of potassa to a solution of a salt of mercury; oxide of mercury will be immediately precipitated. If the mercury in the liquid shall have been in the form of a protoxide, the precipitate will be of a dark colour, but when it contains the peroxide alone, the precipitate has sometimes a reddish colour but is generally yellow, the peroxide disengaged combining with a portion of water and forming a hydrate. Collect the precipitate on a filter, dry it, and expose it to heat at the bottom of a small test tube over a spirit lamp, when globules of metallic mercury will be seen.

Put a small quantity of a solution of a salt of the protoxide of mercury into a glass, and fill it up with lime water; protoxide of mercury will be immediately thrown down and give a dark colour to the liquid.

Into another glass, put a similar quantity of a solution of a salt of the peroxide of mercury, and add lime water as before; the solution will become of a yellow colour from the separation of peroxide of mercury in combination with water.

987. Add some hydrosulphuret of ammonia or pass a stream of sulphuretted hydrogen gas through a diluted solution of a mercurial salt, a copious black precipitate will be thrown down of sulphuret of mercury, probably in combination with water.

988. Add a solution of the muriate of tin (muriate of the protoxide) to a solution of a salt of the protoxide of mercury; a copious ash-grey coloured precipitate will immediately appear, consisting principally of metallic mercury, the oxide of tin combining with the oxygen with which the mercury was previously united, and passing to a higher state of oxidation.

989. A solution of the ferrocyanate of potassa gives a white precipitate with salts of mercury; muriatic acid and solutions of the muriates give a white precipitate with salts of the protoxide, composed of the chloride of mercury.

990. All the salts of mercury are completely decomposed or volatilized by exposure to a dull red heat." 337.

The very full and perspicuous account of the tests employed to detect the presence of arsenic and its salts, and of the best mode of employing these, is extremely well worth a most attentive examination by the medical student. We cannot afford room to enlarge upon this subject at present, but may probably, at an early opportunity, revert to it; as it is only by having their attention repeatedly drawn to it in the pages of a periodical work, that the great body of the medical profession can retain the facts in their memory.

As every fact connected with toxicological chemistry is important to know, we are induced to extract the following observations on the appropriate treatment in cases of poisoning from opium;—they embrace, in a small compass, all that requires to be done by the medical practitioner.

" 1180. With respect to the antidotes to be employed when an over dose of opium has been given, there is no chemical remedy on which we can depend; the carbonate of potassa has been proposed to precipitate the morphia from its solution, and prevent it from acting so energetically as it otherwise would do. Recourse should always be had to the stomach-pump when it can be procured, and powerful emetics should be administered till vomiting is excited, giving at the same time large quantities of warm water, and preventing the individual from falling into a state of sleep or stupor if possible, by forcing him to walk up and down till its effects have passed off. Diffusible stimuli should be given at the same time, taking care to avoid every thing which may render the morphia more soluble, as vinegar or acids, which would only increase its deleterious effects; though, when the poison has been completely removed, some of them assist materially in restoring the tone of the whole system." 385.

The chapter on the use of the blow-pipe is one which merits and will amply repay the perusal, as a knowledge of this most simple, yet efficient instrument of analysis, is indispensible to the practical chemist. Few persons can avail themselves of its employment, from not understanding the method of continuing the blast for a sufficient length of time.

The directions given by Mr. Reid, we know, from our own experience, will, if followed, enable any one to acquire the power.

"1361. The first thing that the student ought to do with the blow-pipe, should be to learn to keep up a continued blast at a candle with his mouth, while he breathes freely through his nostrils. For this purpose, he must, in the first place, distend his cheeks like a trumpeter, and breathe solely through his nostrils, never allowing his cheeks to collapse. When he can do this easily, he should put a blow-pipe with a very small aperture into his mouth, placing the pointed extremity in the flame of a candle; during expiration, a small part of the expired air will pass through the tube, and during inspiration, also, air will continue to pass through the tube, if he shall have made his cheeks sufficiently tense and elastic, by distending them previously with air, and the same process goes on at each successive expiration and inspiration. Such is the mechanical rule for learning the method of keeping up a continued blast of air with this instrument, which I have seldom seen fail in enabling the beginner to acquire this art in five or ten minutes, and as considerable practice is necessary before he can be able to use it freely, he cannot commence too early with it. At first, he should take it up frequently at intervals of an hour or two, and not continue working with it for more than a quarter of an hour; he will soon, however, learn to keep up a continued blast without any difficulty, or distending his cheeks so much as to make it feel tiresome. It is necessary, indeed, to do so, only when he begins, as he will not otherwise be able to acquire the method of blowing speedily.

1362. During inspiration, the passages connecting the mouth with the lungs and the nostrils, are completely closed, and the blast kept up solely by the air, from the reservoir within the cheeks, from which it is pressed out by the contraction of the muscles, and the stock renewed at each successive expiration." 461.

We must now take leave of Mr. Reid; and in doing so, express our hopes that his very valuable work will be appreciated by the lovers of chemistry, as it ought to be. It gives us much pleasure to hear that so many students of the Edinburgh School devote their attention to analytic pursuits; and trust that their example will be followed at the University of London, and at the King's College. Much good has been expected from these rival institutions, and much may be done, if a proper system of practical education be vigorously pursued.

P. S.—After this sheet went to press, we received the second edition of the above valuable work, considerably improved. All the diagrams are renewed on an improved plan, and a full explanation of them is given in the Chapter on Chemical Equivalents. A number of wood-cuts are also added.

